# Peripheral CCL2 induces inflammatory pain via regulation of *I*_h_ currents in small diameter DRG neurons

**DOI:** 10.3389/fnmol.2023.1144614

**Published:** 2023-10-04

**Authors:** Lamei Li, Yuanying Liu, Wenchao Hu, Jing Yang, Suibin Ma, Zhicheng Tian, Zixuan Cao, Kunqing Pan, Ming Jiang, Xia Liu, Shengxi Wu, Ceng Luo, Rou-Gang Xie

**Affiliations:** ^1^Department of Neurobiology, School of Basic Medicine, Fourth Military Medical University, Xi’an, China; ^2^School of Life Sciences & Research Center for Resource Peptide Drugs, Shaanxi Engineering & Technological Research Center for Conversation & Utilization of Regional Biological Resources, Yan’an University, Yan’an, China; ^3^Heart Hospital, Xi’an International Medical Center Hospital, Xi’an, China; ^4^No.6 Cadet Regiment, School of Basic Medical Sciences, Fourth Military Medical University, Xi’an, China; ^5^No.19 Cadet Regiment, School of Basic Medical Sciences, Fourth Military Medical University, Xi’an, China

**Keywords:** the C-C motif chemokine ligand 2 (CCL2), the C-C motif chemokine receptor 2 (CCR2), dorsal root ganglion (DRG), hyperpolarization-activated current (IH), nociceptor, Pain

## Abstract

The C-C motif chemokine ligand 2 (CCL2) has been implicated in chronic pain, but its exact mechanism of peripheral sensitization is unknown. In this study, we aimed to clarify the mechanism of CCL2 regulation of ion channels. Our behavioral experiments revealed that ZD7288, a blocker of *I*_h_ current, can inhibit CFA and CCL2-mediated mechanical and thermal nociceptive sensitization. Furthermore, patch clamp studies demonstrated that CFA-induced peripheral sensitization primarily affects the excitability of small-diameter DRG neurons. Further studies revealed that inflammatory pain caused by CFA or incubation of DRG with CCL2 mainly affected *I*_h_ currents in small-diameter DRG neurons, which were blocked by co-incubation CCR2 antagonist INCB3344 or adenylate cyclase inhibitor SQ22536. Immunohistochemical staining showed that both intraplantar injection of CFA as well as DRG injection of CCL2 resulted in significant upregulation of CCR2^+^/HCN2^+^ expression. In conclusion, we suggest in the inflammatory pain state, CCL2 can act on small-diameter DRG neurons, leading to upregulation of HCN2 expression and consequently *I*_h_, which in turn leads to neuronal hyperexcitability.

## Introduction

Peripheral tissue injury/inflammation often leads to chronic pain. Peripheral sensitization due to hyperexcitability of dorsal root ganglion (DRG) neurons and central sensitization of spinal cord neurons are important reasons for the prolongation of chronic pain. There is growing evidence that inflammation-induced central sensitization is due in part to increased C-fiber afferents caused by spontaneous activity of C-nociceptors (Latremoliere and Woolf, 2009; Kleggetveit et al., 2012; Weng et al., 2012; Hsieh et al., 2015). However, the ion channel mechanisms and underlying molecular mechanisms of nociceptive receptor hyperexcitability that are relevant to chronic inflammatory pain are not fully understood.

The C-C motif chemokine ligand 2 (CCL2), also known as monocyte chemotactic protein 1 (MCP-1), which regulates the migration of monocytes into tissues. A growing number of studies have shown that CCL2 upregulation mediates increased DRG excitability (Gao et al., 2009; Kao et al., 2012; Liu et al., 2016; Dansereau et al., 2021). Long non-coding RNAs (lncRNAs) nerve injury-specific lncRNAs (NIS-lncRNAs) can lead to nociceptive sensitization by regulating the upregulation of CCL2 on DRG (Du et al., 2022). CCL2/CCR2 performs an essential role in the development of peripheral arthritis pain (Ramesh, 2014; Llorián-Salvador et al., 2016; Dansereau et al., 2021). According to Zhang and De Koninck, after ganglion ligation, there was an increase in CCL2 expression in neurons with small to large diameters that express the nerve injury marker ATF-3 (Zhang and De Koninck, 2006). The results suggest that injured neurons exhibit an increased expression of CCL2. CCL2 can act directly on DRG neurons, resulting in increased neuronal firing (White et al., 2005; Wang et al., 2010), however the causes of the increased neuronal firing associated with CCL2 need to be further investigated.

Previous studies demonstrated that CCL2 can regulate synaptic plasticity by interacting with CCR2 on lamina IIo excitatory neurons, suggests that CCL2 may be involved in the development of central sensitization in chronic pain (Biber and Boddeke, 2014; Xie et al., 2018b). In addition, we find that CCL2 directly interacts with CCR2, enhancing NMDAR-induced currents, eventually leading to inflammatory pain. This is mainly achieved through the CCL2-CCR2-pERK-GluN2B pathway (Zhang et al., 2020). And we further demonstrate that CCL2 interacts with presynaptic CCR2 in spinal nociceptor terminals to facilitate spinal synaptic transmission and pain (Ma et al., 2020). However, a number of issues still need to be clarified. For examples, does CCL2 directly act on DRG nociceptor to modulate ion channels variations on neurons? What ion channels are modulated by CCL2/CCR2 leading to DRG hyperexcitability?

Hyperpolarization-activated cation channels (HCNs) activation occurs near the resting membrane potential and activation of HCN channels leads to a mixed non-selective cation flow for depolarizing inward currents (*I*_h_). *I*_h_ partially counteracts the effects of after hyperpolarization (Baker et al., 1987), which leads to higher firing rates and therefore is considered to be the neuronal pacemaker (Wickenden et al., 2009). Numerous studies have shown that HCN2 in peripheral sensory neurons is involved in the development of chronic pain. Chronic inflammatory pain has been associated with an increase in excitability due to *I*_h_ (Song et al., 2012; Weng et al., 2012; Jansen et al., 2021). Studies have shown that HCNs is involved in the regulation of chronic pain in small diameter neurons of the DRG (Weng et al., 2012; Forster et al., 2020) and also in brain (Gao et al., 2022; Yan et al., 2023). The HCN2 ion channel in peripheral primary sensory neurons plays a central role in inflammatory pain and neuropathic pain dependent on prostaglandines and cAMP-increasing effect (Gelderblom et al., 1987; Emery et al., 2011). And peripheral blockade of HCNs rapidly suppresses inflammatory and neuropathic pain (Young et al., 2014). However, it is still not well understood which inflammatory agents are involved in the process of inflammatory pain leading to the upregulation of HCN2.

In this study, we demonstrated that CCL2 can directly act on the CCR2 receptors located in small diameter neurons of the DRG, and leading to up-regulation of HCN2 and hyperpolarization-activated current (*I*_h_) and participate in the formation of DRG hyperexcitability by patch clamp recordings, immunohistochemistry, and behavioral tests.

## Materials and methods

### Animals and pain models

C57BL/6 background wild-type (WT) mice were purchased from the Experimental Animal Center of the Fourth Military Medical University. Adult C57BL/6 mice (male, 6–8 weeks old) were used for electrophysiological studies in dorsal root ganglion (DRG), and behavioral surveys. All animal procedures were approved by the Animal Care Committee of the Fourth Military Medical University, ensuring that all animals were treated humanely and ethically. To establish a chronic inflammatory pain model, we injected Complete Freund’s Adjuvant (CFA, 20 μL, 1 mg/mL, Sigma, St Louis, MO, United States) into the plantar surface of left hind paw to induce inflammatory pain.

### Drugs and administration

CCL2 was from R&D Systems (Minneapolis, MN). ZD7288, *I*_h_ antagonist, was from PlantChemMed Co., Ltd. (Shanghai, China). RS504393, a potent and selective antagonist of CCR2, was from Tocris Bioscience (Bristol, United Kingdom).

### DRG injection of CCL2 and ZD7288

C57BL/6 mice were anaesthetized with 2% isoflurane. The procedure for DRG injection of CCL2 and ZD7288 was the same as previously described (Luo et al., 2017; Ma et al., 2020; Wang et al., 2021). Expose the bilateral iliac crests of the mice to locate the L4 and L5 vertebrae, use the 26-gauge needle mated to a Hamilton syringe (Hamilton, Reno, NV) was inserted at a 45° angle into the lower margin and the intersection of the paravertebral line of the same side L4 and L5 vertebrae, and permeate CCL2 (100 ng/mL) and/or ZD7288 (100 μM, 200 μM) into the intervertebral foramen. When the syringe needle enters the intervertebral foramen, there is a sense of constraint, and the paw retraction reaction of mice is a sign of the needle entering the intervertebral foramen. CCL2 was dissolved in a 1% DMSO/saline mix. ZD7288 was dissolved in saline.

### Behavioral analysis

All mice were acclimated to the testing environment for at least 2 days prior to each test, and acclimated to the individual testing chamber for at least 1 h. All testing was conducted in a blinded manner, ensuring that the results were unbiased and accurate. Mechanical allodynia measure (von Frey test) was conducted using von Frey filaments ranging from 0.008 to 2.000 g (Danmic Global, CA), applied to the plantar skin with sufficient force to bend the fiber and held for 1 s. The paw withdrawal response frequency (the percentage of positive responses to the stimulus) was recorded for each filament, which was applied five times. The mechanical threshold was defined as the force of a specified filament required to elicit an 80% frequency of paw withdrawal. Thermal Hyperalgesia was assessed using a device (IITC model 400, Woodland Hills, CA) specifically designed to measure the latency of paw withdrawal in response to heat stimulation. The mouse was placed on a 2 mm thick thermostatically controlled (30°C) glass plate and stimulated with a beam of radiant heat source on the lateral plantar surface of the hind paw. The duration of time between the initiation of the stimulus and the paw withdrawal was defined as the paw withdrawal thermal latency. The hind paw of each mouse was tested every 5 min, 5 times in total, and the mean of the latency values was calculated.

### Immunofluorescence labelling and confocal analysis

The DRG immunofluorescence staining methods was conducted as previously described (Liu et al., 2015; Han et al., 2021). DRG was sampled at 24 h after intraplantar injection of CFA and 5 h after DRG injection of CCL2. Mice were anesthetized by intraperitoneal (I.p.) injection of 1% pentobarbital sodium and transcardially perfused with phosphate buffer solution (PBS, 20 mL) + 4% paraformaldehyde (PFA, 20 mL). The left L4 and L5 DRGs were removed, postfixed overnight in 4% paraformaldehyde, and cryoprotected in 30% sucrose at 4°C until the tissue sank to the bottom of the container. DRG sections (16 μm) was cut on a cryostat. Briefly, the sections were incubated with a solution containing 0.3% Triton X-100 and 1% bovine serum albumin (BSA) for 3 h at room temperature and were immunostained with primary antibodies including goat-anti-CCR2 (1:150, ThermoFisher, PA1-21623), rabbit-anti-CCR2 (1:500, Novus Biologicals, NBP1-48337), rabbit-anti-HCN2 (1:300, Almone Labs, APC-030) (Hammelmann et al., 2011), goat-anti-CGRP (1:100, Abcam, ab36001) (Tao et al., 2022; Yue et al., 2022) at 4°C overnight. Secondary antibodies including Alexa Fluor 488 (donkey anti-rabbit IgG, 1:500, Abcam, ab150073) and Alexa Fluor 594 (donkey anti-goat IgG, 1:500, Abcam, ab150132), were incubated at room temperature for 4 h. All images were captured by Olympus confocal microscope (Olympus FV3000, Japan), and 20× objectives (NA = 0.8) were used. The images were generated with a 1,024 × 1,024 pixels frame resolution, and the confocal pinhole was 120 μm. Captured images with a 20× magnification under this confocal microscope were processed using Image J, and quantification of CCR2 and merge cells counts were performed manually from 3 DRGs from 3 animals. Analyze the distribution of all neurons. All counting experiments were conducted blinded to the experimental group. Cell diameter measurement is generated by drawing cell profiles through the built-in measurement tool of Olympus cellSens imaging software (Olympus, Japan).

### Western blotting

The L3–L5 DRGs were rapidly homogenized and lysed in RIPA lysis buffer on ice. The protein concentration was determined with a BCA protein assay kit (Thermo Scientific, United States). The protein was electrophoresed on a 10% sodium dodecyl sulfate polyacrylamide gel and then transferred to polyvinyl difluoride (PVDF) membranes (Bio-Rad, Hercules, CA, United States). The PVDF membranes were incubated overnight at 4°C with primary rabbit-anti-CCR2 (1:500, Novus Biologicals, NBP1-48337), rabbit-anti-HCN2 (1:300, Almone Labs, APC-030) or mouse-anti-β-actin (1:5000, Abcam, ab8227) and were incubated with the secondary antibody Donkey anti-Rabbit (1:4000, bioss, bs-0295D) or anti-mouse IgG (1:4000, Cell Signaling Technology, #7056) at ambient temperature for 2 h. An appropriate amount of ZETA luminescent solution was taken, and the corresponding images were saved using a Bio-Rad luminometer for luminescence the protein bands were quantified using ImageJ software (National Institutes of Health, Bethesda, MD, United States).

### Quantitative real time RT-PCR

The DRG of L3–L5 segment was collected from mice in each group (L4–L5 was collected in CCL2 group). RNA was extracted with TRIzol reagent (Invitgen, Carlsad, CA) and reverse transcribed into cDNA with PrimeScript™ RT reagent kit (Takara, Japan). Then TB Green Premix Ex Taq™ II (Takara, Japan) is used for quantitative real-time RT-PCR. The reaction was carried out in StepOne and StepOnePlus Real-Time PCR Systems (Thermo Fisher, United States). In order to quantify, the number of target genes is expressed relative to the number of reference genes (GAPDH) to obtain the normalized target expression value.

Primer sequences were as follows:

HCN2-F GGGAATCGACTCCGAGGTCTAC.

HCN2-R AGACTGAGGATCTTGGTGAAACG.

GAPDH-F TGTGTCCGTCGTGGATCTGA.

GAPDH-R TTGCTGTTGAAGTCGCAGGAG.

### Intact whole mount DRG preparations

The control mice or CFA-inflamed mice (24 h after intraplantar injection of CFA) were anesthetized by intraperitoneal injection of 1% pentobarbital sodium. L4 and L5 DRG were carefully removed and placed in artificial cerebrospinal fluid (ACSF) mixed with ice and water. Carefully peel off the connective tissue and capsule around the DRG, digested with a mixture of 1.0 mg/mL protease and 1.6 mg/mL collagenase for 20 min at 37°C. To further validate the role of chemokine CCL2 on DRG under inflammatory pain, whole mount DRG was prepared and incubated in 100 ng/mL CCL2 for 1, 3, 5 h to permit CCL2 sufficiently influence DRG. The tissues were incubated in ACSF with 95% O_2_ and 5% CO_2_ for at least 1 h before recording.

### Whole cell patch-clamp recording

DRG neurons were observed with 40× objective lens and sealed visually. Pull the patch pipette (5–8 MΩ) out of the borosilicate glass capillaries with a P-97 puller. The ACSF contained (in mM): NaCl 125, KCl 2.5, NaH2PO_4_ 1.2, MgSO_4_ 1.3, CaCl_2_ 2.4, NaHCO_3_ 26, Glucose 15. The intrapipette solutions contained (in mM): KCl 18, K-Glu 132, CaCl_2_ 1, MgCl_2_ 2, EGTA 1.1, HEPES 10, Mg-ATP 2.5, pH 7.4. Osmolarity was adjusted to 290–300 mOsm. Data were obtained by a Digidata 1550B acquisition system (Molecular Devices, United States) and Axopatch 700B amplifier (Molecular Devices, United States) using pCLAMP 10.0 software. Signals were filtered at 5 kHz low-pass, suspended at 10 kHz and stored in a computer hard drive for offline correction and analysis. The compensation of the liquid junction potential is performed by real-time adjustment of the Multiclamp 700B Commander software. The excitability characteristics of neurons were recorded under current clamp, the pattern and the number of action potentials discharged were determined by injecting a series of depolarizing current steps (500 ms duration) and depolarizing current ramp (1,000 ms duration). The minimum current required to evoke the first action potential (rheobase) was determined by current injection steps (10 pA increments).

### *I*_h_ current analysis

*I*_h_ recording was as described previously (Liu et al., 2015; Xie et al., 2018a). Under voltage clamp mode, the membrane potential was held at −60 mV and *I*_h_ current was elicited by hyperpolarizing the holding potentials from −60 to −120 mV in 10 mV increments for a duration of 3 s. For each neuron, measure *I*_h_ by subtracting the initial inward current from the steady-state current. Steady-state inactivation of *I*_h_ was investigated using tail-current analysis.

### Statistics

All data were presented as mean ± SEM. Statistical analysis was conducted using Student’s *t*-test or analysis of variance (ANOVA), followed by either a *post hoc* Fisher’s test or Dunnett’s test. Statistical significance was determined at *p* < 0.05.

## Results

### Intra-ganglionic administration of ZD7288 can attenuate the mechanical and thermal hyperalgesia induced by CFA and CCL2

**Figure 1:**
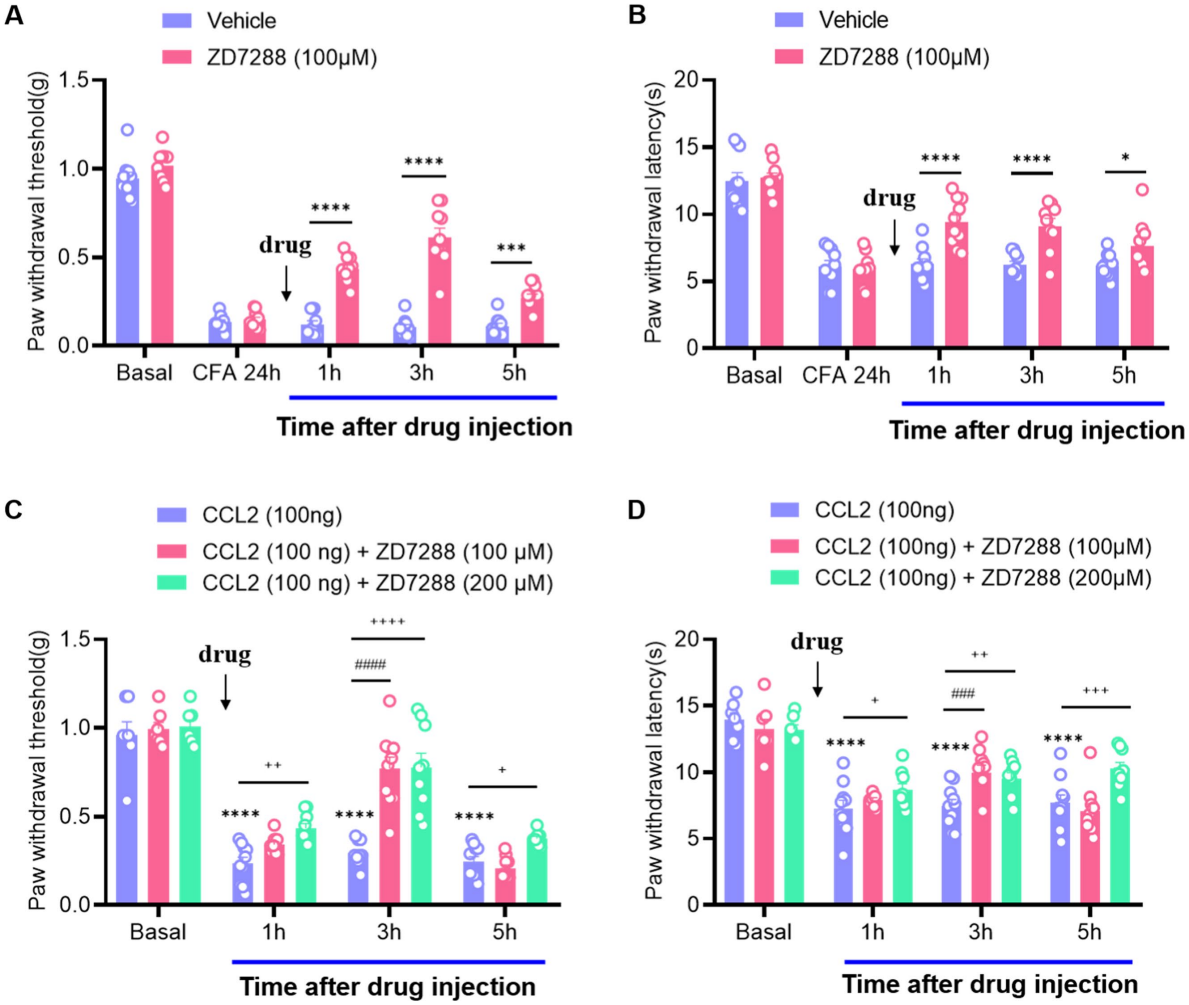
Inhibition of CCL2 and CFA-induced mechanical hyperalgesia and thermal hyperalgesia by intervertebral foramen injection of ZD7288. **(A)** Reversal of CFA-induced mechanical hyperalgesia by ZD7288 (100 μM) given 24 h after CFA injection. **(B)** Reversal of CFA-induced thermal hyperalgesia by ZD7288 (100 μM) given 24 h after CFA injection. **(C)** Prevention of DRG injection CCL2 (100 ng) – induced mechanical hyperalgesia by ZD7288 (100 μM, 200 μM, intervertebral foramen injection). **(D)** Reversal of CCL2-induced heat hyperalgesia by ZD7288 (100 μM, 200 μM, intervertebral foramen injection). All data are presented as mean ± SEM. ^*^ and ^+^ and ^#^ *p* < 0.05, ** and ^++^, and ^##^ *p* < 0.01, ^***^ and ^+++^, and ^###^ *p* < 0.001, ^****^ and ^++++^, and ^####^ *p* < 0.0001, *n* = 9–10 mice/group.

As neuroinflammation leads to hyperexcitation of DRG neurons (Huang et al., 2012; Li et al., 2016), which often induces multiple neuronal discharges, we focused on ion channels responsible for the generation of multiple firing by hyperexcitable neurons. Hyperpolarization-activated current (*I*_h_), as a pacemaker for neuronal excitation, can be activated under hyperpolarization and lead to increased neuronal excitability (Djouhri et al., 2015; Liu et al., 2015). After intraplanar injection of CFA 24 h, a marked increase in mechanical and thermal hyperalgesia was observed, while intervertebral foramen injection of ZD7288 (hyperpolarization-activated cyclic nucleotide-gated (HCN) channel blocker, 100 μM) potently abolished this hyperalgesia and last for 5 h (Figures 1A,B). It is suggested that *I*_h_ produces a significant regulatory role in maintaining inflammatory pain, which is consistent with the previous results (Song et al., 2012; Weng et al., 2012; Jansen et al., 2021). To verify the effect of CCL2 on inflammatory pain, we injected CCL2 in the DRG, which significantly induced a decrease of the mechanical retraction reflex threshold as well as a shortening of the thermal retraction latency in the animals (Figures 1C,D). This hyperalgesia was dose-dependently attenuated by ganglionic injection of ZD7288 (100 μM, 200 μM) (Figures 1C,D). Our results showed that the analgesic effect of ZD7288 in DRG started at 1 h after injection, and the effect peaked at 3 h after injection and could be maintained for more than 5 h.

### CFA-induced inflammation increases excitability of small-diameter DRG neurons but not large or medium-diameter DRG neurons

**Figure 2:**
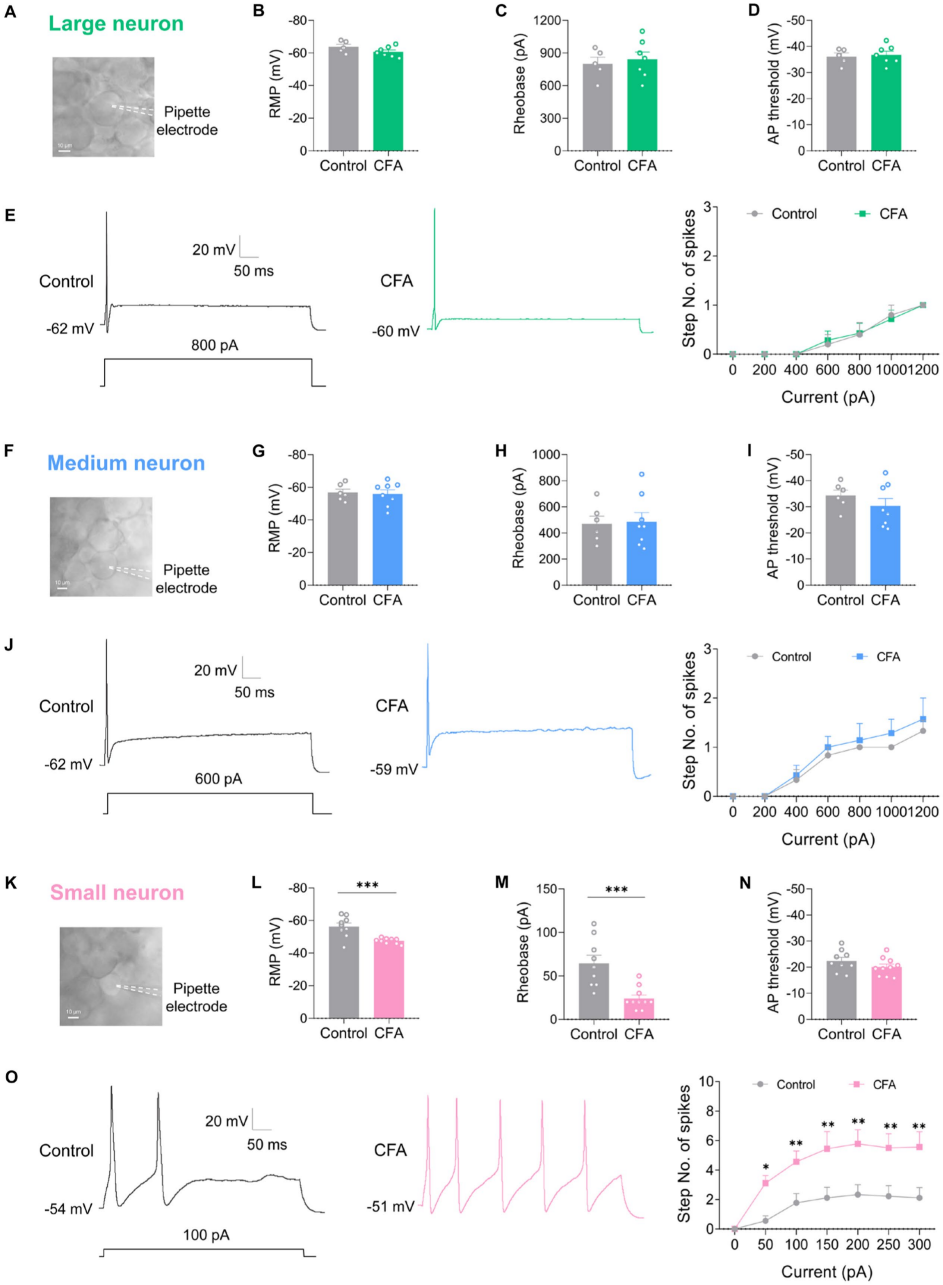
CFA-induced inflammation increases excitability of small-diameter DRG neurons but not large or medium-diameter DRG neurons. **(A,F,K)** Image of recording from a large-diameter (>30 μm), medium-diameter (>20 μm and <30 μm), and small-diameter (<20 μm) DRG neurons in a whole mount DRG preparation are recorded. **(B,G,L)** Resting membrane potential (RMP) of large (**B**, *n* = 5–7), medium (**G**, *n* = 6–8), and small (**L**, *n* = 9–10) DRG neurons in both control and CFA-inflamed states (by unpaired *t*-test). **(C,H,M)** Action potential rheobase of large (**C**, *n* = 5–7), medium (**H**, *n* = 6–8), and small (**M**, *n* = 9–10) DRG neurons in both control and CFA-inflamed states (by unpaired *t*-test). **(D,I,N)** Action potential threshold of large (**D**, *n* = 5–7), medium (**I**, *n* = 6–8), and small (**N**, *n* = 9–10) DRG neurons in both control and CFA-inflamed states (by unpaired *t*-test). **(E,J,O)** Representative traces and I-O curve of large (**E**, *n* = 5–7), medium (**K**, *n* = 6–7), and small (**Q**, *n* = 9) DRG neurons in response to a depolarizing current step in both control and CFA-inflamed states (by two-way ANOVA, Fisher’s LSD test). All data are presented as mean ± SEM. *p < 0.05, **p < 0.01, ***p < 0.001, and ****p < 0.0001.

DRG neurons can be divided into large-diameter (>30 μm) DRG neurons, medium-diameter (>20 μm and <30 μm) DRG neurons, and small-diameter (<20 μm) DRG neurons according to their diameters, corresponding to Aβ, Aδ, and C fibers, respectively. To detect the changes of excitability of the DRG neurons after CFA injection, we made patch-clamp recordings from large-diameter (Figure 2A), medium-diameter (Figure 2F), and small-diameter (Figure 2K) DRG neurons in whole-mount DRG preparations from control and CFA-inflamed mice. Resting membrane potential (RMP) (Figures 2B,G), membrane resistance (Rm) (Table 1), and membrane capacitance (Cm) (Table 1) were comparable on large-diameter, medium-diameter DRG neurons in both control and CFA-inflamed groups. After CFA inflammation, small-diameter DRG neurons exhibited a significant shift towards depolarization in RMP (Figure 2L), Rm and Cm (Table 1), did not differ between the control and CFA-inflamed state. In addition, the rheobase was significant decreased after CFA-inflamed mice vs. control mice in small-diameter DRG neurons (Figure 2M) but not in large-diameter or medium-diameter DRG neurons (Figures 2C,H) and while AP threshold (Figures 2D,I,N) was no difference in CFA-inflamed vs. control groups in large-diameter, medium-diameter and small DRG neurons.

**Table 1:** Passive membrane properties and active membrane properties in three types of DRG neurons.

Type	State	Cm (pF)	Rm (MΩ)	AP Amplitude (mV)	AP half-width (ms)
Large	Control	71.91 ± 3.25	49.24 ± 4.40	102.51 ± 3.18	0.80 ± 0.03
	CFA	76.95 ± 1.63	47.96 ± 4.36	99.71 ± 2.19	1.10 ± 0.07
Medium	Control	39.31 ± 0.80	250.55 ± 21.80	95.55 ± 2.35	1.14 ± 0.04
	CFA	41.29 ± 1.41	192.76 ± 13.56	101.76 ± 1.73	0.91 ± 0.04
Small	Control	24.42 ± 0.33	510.48 ± 29.70	112.75 ± 1.56	4.40 ± 0.09
	CFA	23.88 ± 0.53	494.64 ± 26.46	103.77 ± 1.11	4.36 ± 0.10

The typical traces of action potentials of large-diameter DRG neurons revealed that the depolarizing current step evoked only one spike in both control and CFA-inflamed groups (Figure 2E). I-O curve revealed that the number of spikes in large-diameter DRG neurons is independent on stimulus intensity application (Figure 2E). Additionally, a current ramp of up to 1,000 pA was found to be insufficient to elicit any response (Supplementary Figures S1A,B).

As compared to large-diameter DRG neurons, medium-diameter DRG neurons exhibited a decreased rheobase in the range of 400–600 pA when exposed to depolarizing current steps and this group of neurons also one spike can be induced by depolarizing current steps, and the intensity of current injection as high as 800 pA or more evoked a maximum of two to three spikes in a few neurons in both control and CFA-inflamed groups (Figure 2J). Apart from depolarizing current steps, a current ramp (at 800–1000 pA) can evoke one to two spikes in most neurons (Supplementary Figures S1C,D).

Upon CFA inflammation, the firing frequency of small-diameter DRG neurons was found to be significantly increased, as evidenced by a marked increase in the number of spikes (Figure 2O) and lowered rheobase (Figure 2M). The inference was further corroborated by a ramp current injection (Supplementary Figures S1E,F), which demonstrated that the excitability of small-diameter DRG neurons in CFA-inflamed states was higher than controls. The above results strongly suggest that DRG hyperexcitability caused by CFA occurs mainly in C-nociceptors, such as DRG small-diameter neurons, this finding is in agreement with prior studies (Weng et al., 2012; Wang et al., 2016; Han et al., 2021).

### CFA-induced inflammation increases *I*_h_ amplitude of small-diameter DRG neurons but not large or medium-diameter DRG neurons

**Figure 3:**
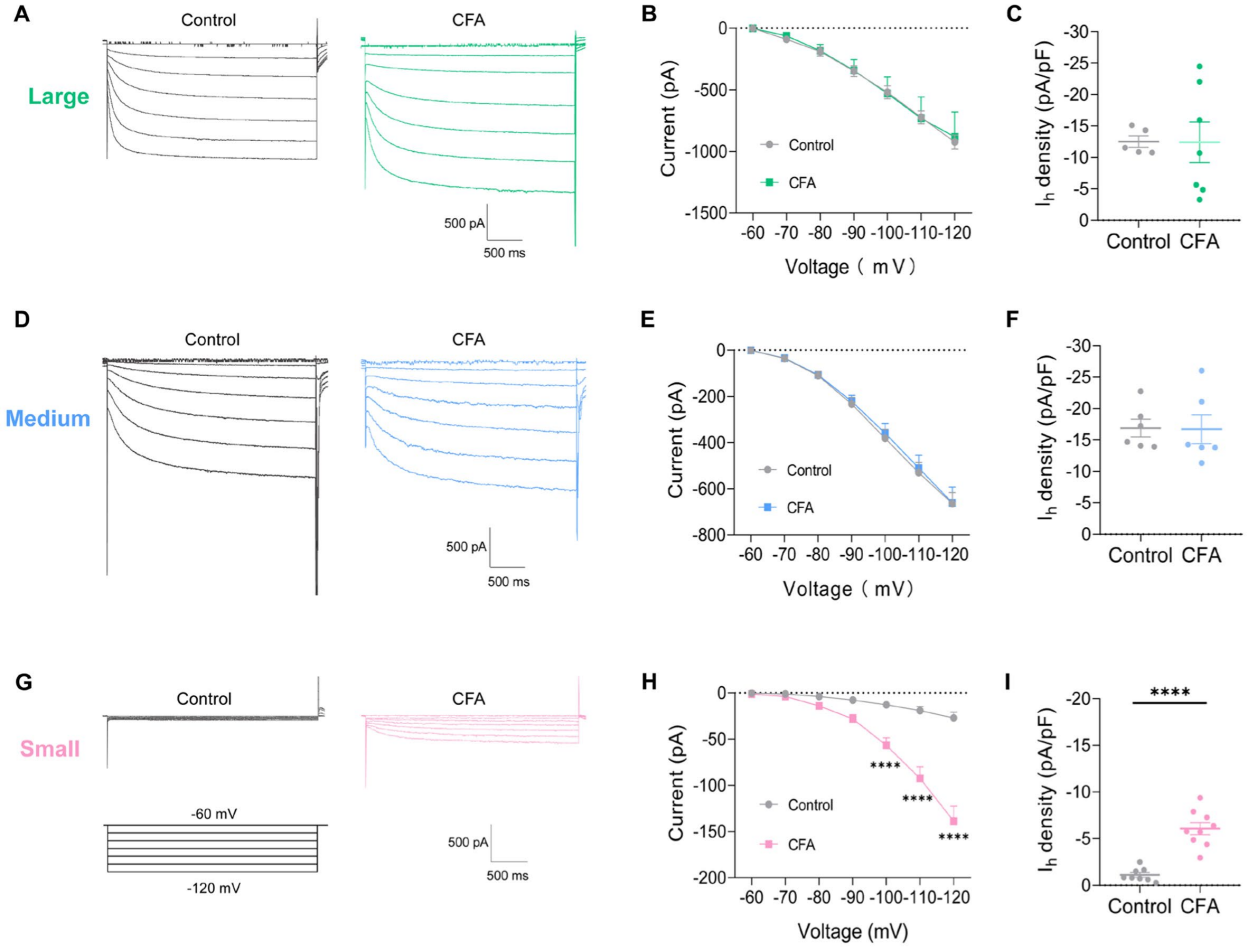
CFA-induced inflammation increases *I*_h_ amplitude of small-diameter DRG neurons but not large-or medium-diameter DRG neurons. **(A,D,G)** Representative traces of *I*_h_ recorded in large- **(A)**, medium- **(D)**, and small-diameter **(G)** DRG neurons in response to hyperpolarizing voltage steps of −60 to −120 mV in 10 mV increments from a holding potential at −60 mV in both control and CFA-inflamed states. The voltage protocol is shown in the lower position of G. **(B,E,H)** Current–voltage relationship of *I*_h_ in large- (**B**, *n* = 5–7), medium- (**D**, *n* = 6), and small-diameter (**F**, *n* = 8–9) DRG neurons from control and CFA-injected mice (by two-way ANOVA, Fisher’s LSD test). **(C,F,I)** *I*_h_ density in large- (**B**, *n* = 5–7), medium- (**D**, *n* = 6), and small-diameter (**F**, *n* = 8–9) DRG neurons from control and CFA-injected mice (by nonparametric Mann–Whitney test), Note the *I*_h_ current densities measured at −120 mV. All data are presented as mean ± SEM. *p < 0.05, **p < 0.01, ***p < 0.001, and ****p < 0.0001.

To further clarify the specific cause of DRG hyperexcitability induced by intraplantar injection of CFA, we further analyzed the *I*_h_ current changes on different types of DRG neurons. Slow inward currents can be induced in DRG neurons by a series of hyperpolarizing step potential from −60 mV to −120 mV in 10 mV increments (Figures 3A,D,G). Both the large-diameter (Figures 3B,C) and medium-diameter (Figures 3E,F) DRG neurons in control and CFA-treated mice revealed no discernible variations in *I*_h_ currents amplitude and *I*_h_ current densities. However, the *I*_h_ current amplitude and *I*_h_ current densities in small-diameter DRG neurons in the CFA-inflamed state were greater than those in control (Figures 3H,I). It is suggested that only the *I*_h_ currents of small-diameter DRG neurons were significantly altered in the CFA-induced inflammatory pain state, which may be related to CFA-induced DRG hyperexcitability, CFA enhances C-nociceptor excitability by activating *I*_h_ currents on small-diameter DRG neurons, thereby mediating peripheral sensitization.

### CCL2-induced inflammation increases excitability and *I*_h_ amplitude of small-diameter DRG neurons

**Figure 4:**
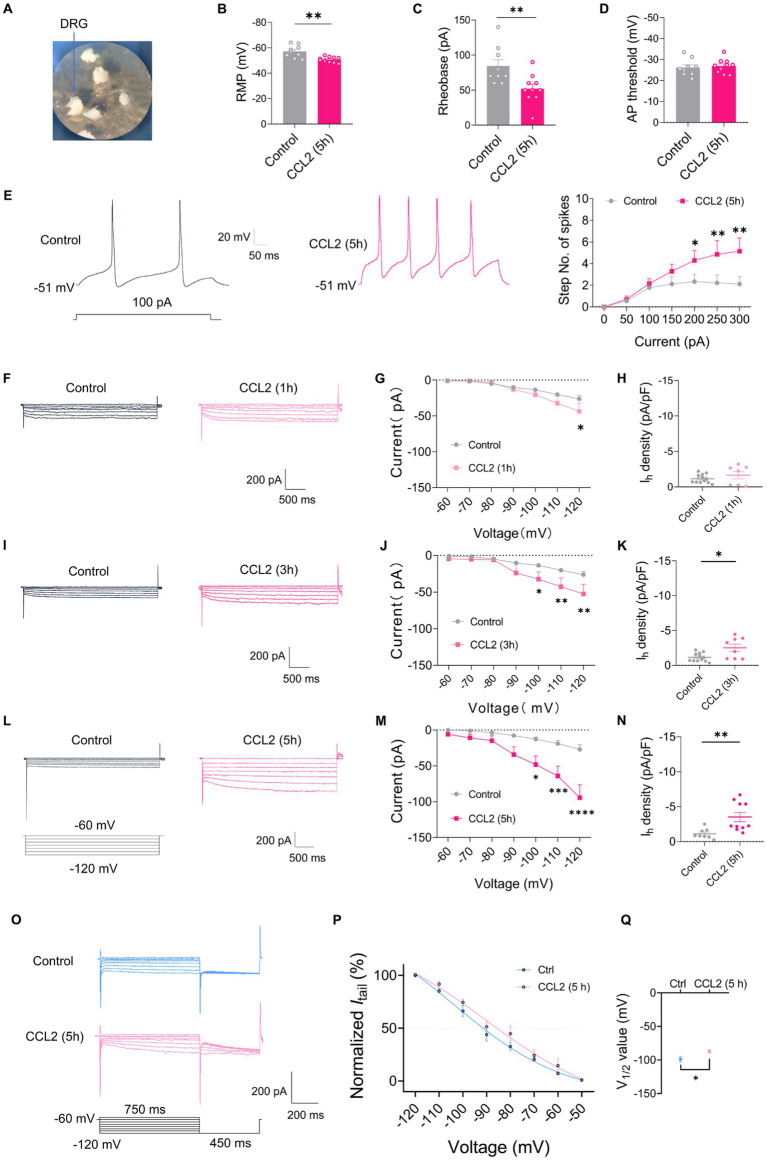
CCL2 incubation leads to elevated excitability and increased *I*_h_ currents in small diameter DRG neurons. **(A)** Showing intact whole mount DRG preparations and incubation of CCL2 (100 ng/mL). **(B–D)** Resting membrane potential (RMP) **(B)**, rheobase **(C)**, and AP threshold **(D)** of small DRG neurons both in control and 5 h after incubation with CCL2 conditions (*n* = 5–7, unpaired *t*-test). **(E)** Representative traces and I-O curve of small DRG neurons in response to a depolarizing current step both in control and 5 h after incubation with CCL2 conditions (*n* = 9, by two-way ANOVA, Fisher’s LSD test). **(F,I,L)** Representative traces of *I*_h_ recorded in small DRG neurons in response to hyperpolarizing voltage steps of −60 to −120 mV in 10 mV increments from a holding potential at −60 mV both in control and 1 h **(F)**, 3 h **(I)**, 5 h **(L)** incubation with CCL2 conditions. The voltage protocol is shown in the lower position of **L**. **(G,J,M)** Current–voltage relationship of *I*_h_ in small DRG neurons from control and 1 h (**G**, *n* = 10–12), 3 h (**J**, *n* = 9–12), 5 h (**M**, *n* = 8–10) incubation with CCL2 (by two-way ANOVA, Fisher’s LSD test). **(H,K,N)** *I*_h_ density in small DRG neurons from control and 1 h (**H**, *n* = 10–12), 3 h (**K**, *n* = 9–12), 5 h (**N**, *n* = 8–10) incubation with CCL2 conditions (by nonparametric Mann–Whitney test). **(O)** The activation curve of *I*_h_ from control and 5 h incubation with CCL2 was constructed by measuring tail currents at −120 mV after application of prepulse potentials between −50 to −120 mV and fitted with a Boltzmann equation. **(P)** The midpoint (V_1/2_) for activation of *I*_h_ in small DRG neurons after incubation with CCL2 5 h was shifted 12 mV in the depolarizing direction compared to control neurons. **(Q)** The difference of V_1/2_ for activation of *I*_h_ between two groups was significant changed. All data are presented as mean ± SEM. *p < 0.05, **p < 0.01, ***p < 0.001, and ****p < 0.0001.

**Figure 5:**
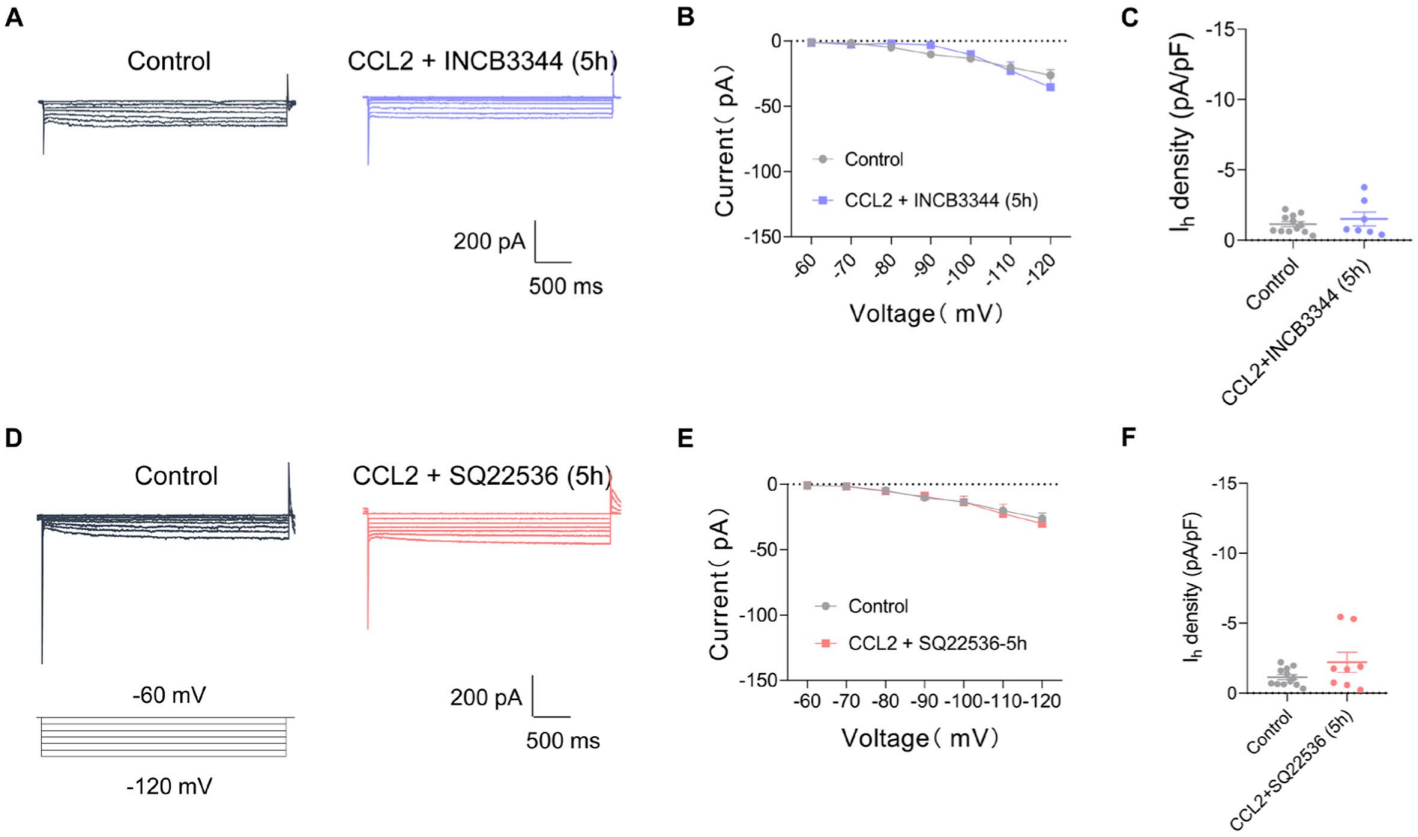
Co-incubation with INCB3344 or SQ22536 eliminated increased *I*_h_ current caused by CCL2 in small diameter DRG neurons. **(A,D)** Representative traces of *I*_h_ recorded in small DRG neurons in response to hyperpolarizing voltage steps of −60 to −120 mV in 10 mV increments from a holding potential at −60 mV in both in control and 5 h incubation with CCL2 and INCB3344 (10 nM) **(A)** or CCL2 and SQ22536 (50 μM) **(D)**. **(B)** Current–voltage relationship of *I*_h_ in small DRG neurons from control and 5 h after incubation with CCL2 and INCB3344 (10 nM) (**B**, *n* = 8–12) or CCL2 and SQ22536 (50 μM) (**E**, *n* = 8–12) (by two-way ANOVA, Fisher’s LSD test). **(C)** *I*_h_ density in small DRG neurons from control and 5 h incubation with CCL2 and INCB3344 (10 nM) (**C**, *n* = 8–12) or CCL2 and SQ22536 (50 μM) (**F**, *n* = 8–12) (by nonparametric Mann–Whitney test). All data are presented as mean ± SEM. *p < 0.05, **p < 0.01, ***p < 0.001, and ****p < 0.0001.

To further validate the role of chemokine CCL2 on DRG under inflammatory pain, whole mount DRG was prepared and incubated in 100 ng/mL CCL2 for 5 h to permit CCL2 to fully influence the DRG (Figure 4A). After incubation CCL2, small-diameter DRG neurons demonstrated significant alterations in membrane characteristics and increased excitability. Resting membrane potential (RMP) (Figure 4B) and rheobase (Figure 4C) exhibited a significant reduction after incubation CCL2 compared to control neurons, while the AP threshold (Figure 4D) remained intact. Enhanced excitability after CCL2 incubation 5 h is manifested as increased number of spikes by step (Figure 4E) and ramp (Supplementary Figures S1G,H) current injection. More importantly, the *I*_h_ current amplitude of small-diameter DRG was much higher after incubation with CCL2 1 h (Figures 4F,G), 3 h (Figures 4I,J), and 5 h (Figures 4L,M) vs. control. The *I*_h_ current densities was significantly greater in CCL2 incubation 3 h (Figure 4K) and 5 h (Figure 4N) than in control condition, but not significant in 1 h (Figure 4H). The activation curve of *I*_h_ was constructed by measuring tail currents (Figure 4O) at −120 mV after application of prepulse potentials between −50 to −120 mV and fitted with a Boltzmann equation (Figure 4P). As shown in Figure 4Q, the midpoint (V_1/2_) for activation of *I*_h_ in the DRG small diameter neurons incubated with CCL2 5 h (−87.1 ± 2.49 mV) was shifted 12 mV in the depolarizing direction compared to control neurons (−98.9 ± 4.24 mV).

To clarify whether the CCL2-mediated increment in *I*_h_ current is mediated by activation of CCR2, the receptor of CCL2, we applied a CCR2 antagonist INCB3344. However, the increase of *I*_h_ current amplitude and *I*_h_ current densities after incubation with CCL2 5 h was inhibited by co-incubation of 10 nM INCB3344 (Figures 5A–C). These results indicate that hyperexcitability and increased *I*_h_ in small-diameter DRG neurons incubated with CCL2 might be mediated by activation of CCL2-CCR2 pathway.

CFA-mediated inflammatory response in mediating the enhancement of *I*_h_ current has been widely studied, and the changes in *I*_h_ current may depend on prostaglandines and cAMP-increasing effect (Gelderblom et al., 1987; Emery et al., 2011). To further clarify the intracellular signaling pathway of CCL2 leading to increased *I*_h_ currents in small diameter neurons of DRG, *I*_h_ currents on DRG small diameter neurons were further detected after 5 h of co-incubation of the adenylate cyclase inhibitor (SQ22536, 50 μM) with CCL2. The adenylate cyclase inhibitor significantly inhibited the increased *I*_h_ current caused by 5 h incubation with CCL2 (Figures 5D–F). It is suggested that CCL2 may act on CCR2 in small-diameter neurons of DRG by enhancing intracellular cAMP signaling pathway, leading to an increase in *I*_h_.

### CFA and CCL2 induces upregulation of DRG CCR2, HCN2, and CGRP

**Figure 6:**
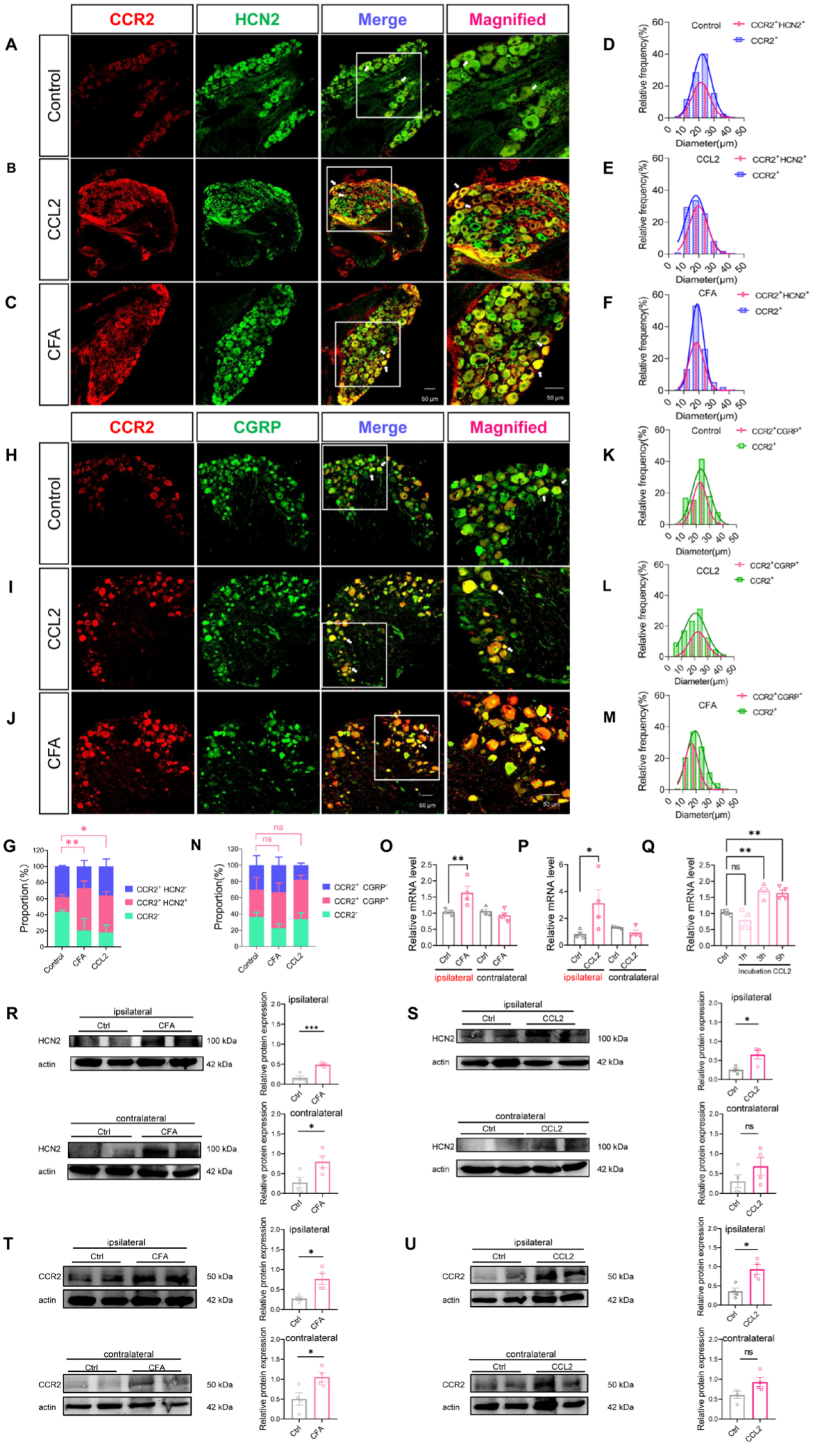
CFA and CCL2 induces upregulation of CCR2, HCN2, and CGRP expression in DRG. **(A–C)** Representative 20× and 40× (magnified) IHC images of CCR2 (red) and HCN2 (green) in DRGs of control, DRG injection of CCL2 and intraplantar CFA injection mice. Scale bar, 50 μm. **(D–F)** Size distribution and CCR2^+^/HCN2^+^ co-expression characterization of CCR2-positive neurons in DRGs of control, intervertebral foramen injection CCL2 and intraplantar CFA injection mice. **(H–J)** Representative 20× and 40× (magnified) IHC images of CGRP (red) and CCR2 (green) in DRGs of control, intervertebral foramen injection CCL2 and intraplantar CFA injection mice. Scale bar, 50 μm. **(K–M)** Size distribution and CCR2^+^/CGRP^+^ co-expression characterization of CCR2-positive neurons in DRGs of control, intervertebral foramen injection CCL2 and intraplantar CFA injection mice. Note that for CFA-inflamed group, ipsilateral L4/L5 DRGs were obtained and analyzed at 24 h after intraplantar CFA injection; for CCL2-inflamed group, ipsilateral L4/L5 DRGs were obtained and analyzed at 5 h after intervertebral foramen injection CCL2. **(G)** Percentage of CCR2^+^/HCN2^−^, CCR2^+^/HCN2^+^, CCR2-cells in the total number of cells. Percentage of CCR2^+^/HCN2^+^ in DRG after intraplantar injection of CFA or DRG injection of CCL2 were significantly increased vs. control mice (by one-way ANOVA, Newan–Keuls test, *n* = 3 per group). **(N)** Percentage of CCR2^+^/CGRP^−^, CCR2^+^/CGRP^+^, CCR2-cells in the total number of cells (*n* = 3 per group). **(O–Q)** The mRNA expression of HCN2 was identified by RT-qPCR in 12 groups (*n* = 6 per group). **(R)** The expression of HCN2 protein in 4 groups of CFA 1 d mice (6 cases in each group) was detected by Western blot. **(S)** The expression of HCN2 protein in 4 groups of DRG injection of CCL2 5 h mice (6 cases in each group) were detected by Western blot. **(T)** The expression of CCR2 protein in 4 groups of CFA 1 d mice (6 cases in each group) was detected by Western blot. **(U)** The expression of CCR2 protein in 4 groups of DRG injection of CCL2 5 h mice (6 cases in each group) were detected by Western blot (by nonparametric Mann–Whitney test). CCL2 represents for DRG injection CCL2 5 h in **B,E,I,L,G,N–P,S–U**. All data are presented as mean ± SEM. *p < 0.05 and **p < 0.01.

In order to further verify that CCL2 enhances *I*_h_ currents in DRG neurons through up-regulation of HCN channels in DRG neurons under inflammatory pain. Immunohistochemical fluorescence techniques were employed to assess the expression of CCR2, HCN2, and CGRP in DRG neurons of control, intraplantar injection of CFA and DRG injection of CCL2 mice (Figures 6A–C,H; Supplementary Figure S2). Confocal analysis revealed the co-staining rates of CCR2 with HCN2 or CGRP are 18.94% (Figure 6D) and 33.72% (Figure 6K), respectively in control state. After DRG injection of CCL2, the proportion of neurons co-labeled with CCR2 and HCN2 or CGRP was increased significantly to 45.98% (Figure 6E) and 48.6% (Figure 6L). After intraplantar injection of CFA, the co-labeled of CCR2 and HCN2 or CGRP increased to 52.54% (Figure 6F) and 44.12% (Figure 6M). The numeration of CCR2^+^/HCN2^+^ neurons (Figure 6G) but not CCR2^+^/CGRP^+^ neurons (Figure 6N) in the DRG was upregulation in both DRG injection of CCL2 and intraplantar CFA injection.

To further clarify the variation of HCN2, western blot experiments were performed as well as PCR experiments. 24 h after intraplantar injection of CFA, HCN2 mRNA was upregulated in the ipsilateral DRG of mice, but the contralateral DRG showed insignificant changes in HCN2 mRNA (Figure 6O). Similarly, 5 h after the DRG was injected with CCL2, HCN2 mRNA was up-regulated in the ipsilateral DRG of mice, but HCN2 mRNA in the contralateral DRG was not changed (Figure 6P). Notably, the HCN2 mRNA of DRG was much higher after incubation with CCL2 for 3 h, and 5 h vs. control (Figure 6Q). Western blot experiments showed that HCN2 protein expression levels were up-regulated in DRG after intraplantar injection of CFA for 24 h and intra-DRG injection of CCL2 for 5 h in ipsilateral side (Figures 6R,S; Supplementary Figures S3A,B). Western blot experiments showed that CCR2 protein expression levels were up-regulated in DRG after intraplantar injection of CFA for 24 h and intra-DRG injection of CCL2 for 5 h in ipsilateral side but not contralateral side (Figures 6T,U; Supplementary Figures S4A,B). The above results suggest that plantar injection of CFA, DRG injection of CCL2 and DRG incubation of CCL2 can all lead to upregulation of HCN2 on DRG.

## Discussion

In our study, we apply an inflammatory pain model to explore potential ion channel mechanisms of CCL2 in DRG, and found that CCL2 can act directly on DRG neurons and cause a decrease in mechanical retraction reflex threshold and a shortening of thermal retraction latency in animals, while DRG injection of ZD7288, a blocker of *I*_h_ current, can inhibit CFA-mediated mechanical and thermal nociceptive sensitization and dose-dependently inhibit mechanical and thermal nociceptive sensitization caused by DRG injection of CCL2. Patch-clamp studies have shown that decreased RMP, reduced rheobase, and increased firing frequency in small-diameter DRG neurons are important manifestations of peripheral sensitization by intraplantar injection of CFA. In the meantime, there was no significant change in the excitability of large-diameter as well as medium-diameter DRG neurons. Further studies showed that inflammatory pain caused by CFA mainly affected *I*_h_ currents in small-diameter DRG neurons, but not in large-or medium-diameter DRG neurons. And co-incubation of DRG with CCL2 significantly increased the excitability of small-diameter DRG neurons, leading to a decrease in RMP, rheobase, and an increase in firing frequency. And CCL2 incubation can significantly increase the *I*_h_ current of small-diameter DRG. Ultimately, immunohistochemical staining showed that both intraplantar injection of CFA as well as DRG injection of CCL2 resulted in significant upregulation of CCR2 expression and an increase from 18.94 to 45.98% as well as 52.54% in CCR2 and HCN2 co-labeled neurons, with CCR2 mainly expressed in CGRP-positive small diameter DRG neurons.

CCR2 has been identified as the primary receptor for CCL2 (also referred to as Monocyte chemoattractant protein-1, MCP-1), a chemokine that plays a key role in the recruitment of pro-inflammatory blood monocytes to sites of tissue inflammation (Deshmane et al., 2009; Chu et al., 2014; Singh et al., 2021). Tanaka et al. reported that CCL2 was rapidly upregulated (less than 4 h) on DRG neurons after partial sciatic nerve ligation (PNSL) (Tanaka et al., 2004). CFA-induced paw edema elicited an upregulation of CCL2/CCR2 expression in ipsilateral DRGs (Luo et al., 2018; Dansereau et al., 2021). CCL2 and CCR2 mRNA was upregulated in 24 h in a mouse model of contact hypersensitivity (Jiang et al., 2019). This strongly suggests the upregulation of CCR2 on DRG is a crucial factor in induction of pain. However, whether the upregulation of CCL2/CCR2 on DRG cells can directly influence ion channels and play a role in periphery sensitization is unclear. Our study further demonstrates that CCL2-CCR2 could directly influence *I*_h_ in small DRG neuron, which may be an important cause of inflammation-induced periphery sensitization.

Numerous studies have shown that direct action of CCL2 on the spinal dorsal horn can mediate central sensitization by enhancing the NMDAR-induced current in the dorsal horn neurons via CCR2 (Sonekatsu et al., 2016; Xie et al., 2018b; Zhang et al., 2020). CCL2 can also act on CCR2 receptors in the presynaptic terminals of the spinal dorsal horn to mediate presynaptic glutamate release (Huang et al., 2014; Ma et al., 2020), leading to central sensitization. It has been shown that intraplantar injections of CCL2 produce nociceptive sensitization to thermal and mechanical stimuli in rats (Qin et al., 2005; Hackel et al., 2013). Upregulation of CCL2 in the L5 DRG by intrathecal injection of AAV5-EF1α-CCL2 enhances neuronal axon regeneration (Kwon et al., 2015; Niemi et al., 2016), however few *in vivo* studies have investigated the effects of exogenous CCL2 directly on the DRG, and our study showed that DRG injection of CCL2 significantly induce mechanical and thermal hyperalgesia, and that this hyperalgesia can be dose-dependently inhibited by the *I*_h_ blocker ZD7288.

Research into the influence of CCL2 on ion channels has primarily centered on NMDA receptors. CCL2/CCR2 signaling mediates chronic pain via regulating NR2B in spinal dorsal horn neurons (Xie et al., 2018b; Zhang et al., 2020). CCL2/CCR2-mediated NMDAR activity in D1R- and D2R-containing neurons after peripheral nerve injury (Wu et al., 2018). Luo et al. verified that CCL2 causes inflammatory pain by regulating Na_V_ channel activity in primary afferent neurons (Zhao et al., 2014; Luo et al., 2018). Patch-clamp recordings of acutely isolated DRG neurons revealed that CCL2 directly depolarized small and medium-sized sensory neurons in CFA-treated rats and enhanced whole-cell TTX-sensitive sodium currents (Zhao et al., 2014; Luo et al., 2018). Chronic inflammatory pain has been associated with an increase in excitability due to hyperpolarization-activated current (*I*_h_) (Song et al., 2012; Weng et al., 2012; Jansen et al., 2021), and the changes in *I*_h_ current may depend on prostaglandines and cAMP-increasing effect (Gelderblom et al., 1987; Emery et al., 2011). However, the mechanism responsible for the elevated *I*_h_ in inflammatory states is still unclear. Our study found that exogenously administered DRG injected with CCL2 for 5 h can significantly upregulate HCN2 and that DRG incubation with CCL2 for 5 h can lead to a significant increase in *I*_h_ which were blocked by co-incubation CCR2 antagonist INCB3344 or adenylate cyclase inhibitor SQ22536. It was further clarified that CCL2 may act on CCR2 in small-diameter DRG neurons in the inflammatory pain state, upregulating HCN2 channels and *I*_h_ through the intracellular cAMP signaling pathway, thus leading to neuronal hyperexcitability. It shows that adenylate cyclase inhibitor SQ22536 can inhibit CCL2-induced Akt phosphorylation (Mizutani et al., 2009). Neutralizing CCL2 inhibits neuronal growth activity in cAMP-treated neuron-macrophage co-cultures conditioned medium (Kwon et al., 2015). It has been shown that cAMP can also act directly on HCN channels, thereby directly increasing *I*_h_ currents (Ulens and Tytgat, 2001; Wainger et al., 2001; Leypold et al., 2019). Whether the effects of CCL2 on small-diameter DRG neurons results in changes in intracellular cAMP that act directly on CCR2 receptors on neurons or whether they originate from other types of cells (e.g., macrophages) remains to be further investigated. Meanwhile, further studies are still needed to investigate the changes of cAMP in DRG neurons during inflammatory pain states.

In conclusion, our results suggest that CCL2 can upregulate HCN2 in specific types of sensory neurons, leading to an increased number of small-diameter DRG neurons discharging in an inflammatory state, which is a potential cause of peripheral sensitization. This study also provides new insights and targets for the peripheral treatment of chronic pain.

## Data availability statement

The original contributions presented in the study are included in the article/Supplementary material, further inquiries can be directed to the corresponding authors.

## Ethics statement

The animal study was approved by the Animal Care Committee of the Fourth Military Medical University. The study was conducted in accordance with the local legislation and institutional requirements.

## Author contributions

LL carried out electrophysiological experiments. LL and WH performed immunostaining experiments. WH performed PCR experiments and analysis. LL and YL carried out behavioral experiments. YL performed the western blot experiments. JY, SM, ZT, ZC and KP performed immunostaining experiments. R-GX, CL, and SW designed, drafted, and finished the final manuscript. MJ and XL reviewed and edited the manuscript. All authors contributed to the article and approved the submitted version.

## References

[ref1] Baker M. Bostock H. Grafe P. Martius P. (1987). Function and distribution of three types of rectifying channel in rat spinal root myelinated axons. J. Physiol. 383, 45–67. doi: 10.1113/jphysiol.1987.sp016395, PMID: 2443652 PMC1183056

[ref2] Biber K. Boddeke E. (2014). Neuronal CC chemokines: the distinct roles of CCL21 and CCL2 in neuropathic pain. Front. Cell. Neurosci. 8:210. doi: 10.3389/fncel.2014.00210 25147499 PMC4124792

[ref3] Chu H. X. Arumugam T. V. Gelderblom M. Magnus T. Drummond G. R. Sobey C. G. (2014). Role of CCR2 in inflammatory conditions of the central nervous system. J. Cereb. Blood Flow Metab. 34, 1425–1429. doi: 10.1038/jcbfm.2014.120, PMID: 24984897 PMC4158674

[ref4] Dansereau M.-A. Midavaine É. Bégin-Lavallée V. Belkouch M. Beaudet N. Longpré J.-M.. (2021). Mechanistic insights into the role of the chemokine CCL2/CCR2 axis in dorsal root ganglia to peripheral inflammation and pain hypersensitivity. J. Neuroinflammation 18:79. doi: 10.1186/s12974-021-02125-y, PMID: 33757529 PMC7986025

[ref5] Deshmane S. L. Kremlev S. Amini S. Sawaya B. E. (2009). Monocyte chemoattractant protein-1 (MCP-1): an overview. J. Interferon. Cytokine Res. Off. J. Int. Soc. Interferon. Cytokine Res. 29, 313–326. doi: 10.1089/jir.2008.0027, PMID: 19441883 PMC2755091

[ref6] Djouhri L. Al Otaibi M. Kahlat K. Smith T. Sathish J. Weng X. (2015). Persistent hindlimb inflammation induces changes in activation properties of hyperpolarization-activated current (*I*_h_) in rat C-fiber nociceptors in vivo. Neuroscience 301, 121–133. doi: 10.1016/j.neuroscience.2015.05.074, PMID: 26047727

[ref7] Du S. Wu S. Feng X. Wang B. Xia S. Liang L.. (2022). A nerve injury-specific long noncoding RNA promotes neuropathic pain by increasing Ccl2 expression. J. Clin. Invest. 132:153563. doi: 10.1172/JCI153563, PMID: 35775484 PMC9246381

[ref8] Emery E. C. Young G. T. Berrocoso E. M. Chen L. McNaughton P. A. (2011). HCN2 ion channels play a central role in inflammatory and neuropathic pain. Science 333, 1462–1466. doi: 10.1126/science.1206243 21903816

[ref9] Forster L. A. Jansen L.-A. R. Rubaharan M. Murphy A. Z. Baro D. J. (2020). Alterations in SUMOylation of the hyperpolarization-activated cyclic nucleotide-gated ion channel 2 during persistent inflammation. Eur. J. Pain 24, 1517–1536. doi: 10.1002/ejp.1606, PMID: 32446289 PMC7496191

[ref10] Gao S.-H. Tao Y. Zhu Y. Huang H. Shen L.-L. Gao C.-Y. (2022). Activation of dopamine D2 receptors alleviates neuronal hyperexcitability in the lateral entorhinal cortex via inhibition of HCN current in a rat model of chronic inflammatory pain. Neurosci. Bull. 38, 1041–1056. doi: 10.1007/s12264-022-00892-z, PMID: 35705785 PMC9468209

[ref11] Gao Y.-J. Zhang L. Samad O. A. Suter M. R. Yasuhiko K. Xu Z.-Z.. (2009). JNK-induced MCP-1 production in spinal cord astrocytes contributes to central sensitization and neuropathic pain. J. Neurosci. Off. J. Soc. Neurosci. 29, 4096–4108. doi: 10.1523/JNEUROSCI.3623-08.2009, PMID: 19339605 PMC2682921

[ref12] Gelderblom H. Reupke H. Winkel T. Kunze R. Pauli G. (1987). MHC-antigens: constituents of the envelopes of human and simian immunodeficiency viruses. Zeitschrift Fur Naturforschung. C. J. Biosci. 42, 1328–1334. doi: 10.1515/znc-1987-11-1230, PMID: 2452527

[ref13] Hackel D. Pflücke D. Neumann A. Viebahn J. Mousa S. Wischmeyer E.. (2013). The connection of monocytes and reactive oxygen species in pain. PLoS One 8:e63564. doi: 10.1371/journal.pone.0063564, PMID: 23658840 PMC3642180

[ref14] Hammelmann V. Zong X. Hofmann F. Michalakis S. Biel M. (2011). The cGMP-dependent protein kinase II is an inhibitory modulator of the hyperpolarization-activated HCN2 channel. PLoS One 6:e17078. doi: 10.1371/journal.pone.0017078, PMID: 21347269 PMC3038938

[ref15] Han W.-J. Ma S.-B. Wu W.-B. Wang F.-D. Cao X.-L. Wang D.-H.. (2021). Tweety-homolog 1 facilitates pain via enhancement of nociceptor excitability and spinal synaptic transmission. Neurosci. Bull. 37, 478–496. doi: 10.1007/s12264-020-00617-0, PMID: 33355899 PMC8055738

[ref16] Hsieh M.-T. Donaldson L. F. Lumb B. M. (2015). Differential contributions of A-and C-nociceptors to primary and secondary inflammatory hypersensitivity in the rat. Pain 156, 1074–1083. doi: 10.1097/j.pain.0000000000000151, PMID: 25760474 PMC4535358

[ref17] Huang C.-Y. Chen Y.-L. Li A. H. Lu J.-C. Wang H.-L. (2014). Minocycline, a microglial inhibitor, blocks spinal CCL2-induced heat hyperalgesia and augmentation of glutamatergic transmission in substantia gelatinosa neurons. J. Neuroinflammation 11:7. doi: 10.1186/1742-2094-11-7, PMID: 24405660 PMC3896825

[ref18] Huang Z.-J. Li H.-C. Liu S. Song X.-J. (2012). Activation of cGMP-PKG signaling pathway contributes to neuronal hyperexcitability and hyperalgesia after in vivo prolonged compression or in vitro acute dissociation of dorsal root ganglion in rats. Sheng Li Xue Bao 64, 563–576. PMID: 23090497

[ref19] Jansen L. A. R. Forster L. A. Smith X. L. Rubaharan M. Murphy A. Z. Baro D. J. (2021). Changes in peripheral HCN2 channels during persistent inflammation. Channels 15, 165–179. doi: 10.1080/19336950.2020.1870086, PMID: 33423595 PMC7808421

[ref20] Jiang H. Cui H. Wang T. Shimada S. G. Sun R. Tan Z.. (2019). CCL2/CCR2 signaling elicits itch-and pain-like behavior in a murine model of allergic contact dermatitis. Brain Behav. Immun. 80, 464–473. doi: 10.1016/j.bbi.2019.04.026, PMID: 30981714

[ref21] Kao D.-J. Li A. H. Chen J.-C. Luo R.-S. Chen Y.-L. Lu J.-C.. (2012). CC chemokine ligand 2 upregulates the current density and expression of TRPV1 channels and Nav1.8 sodium channels in dorsal root ganglion neurons. J. Neuroinflammation 9:189. doi: 10.1186/1742-2094-9-189 PMID: 22870919 PMC3458897

[ref22] Kleggetveit I. P. Namer B. Schmidt R. Helås T. Rückel M. Ørstavik K.. (2012). High spontaneous activity of C-nociceptors in painful polyneuropathy. Pain 153, 2040–2047. doi: 10.1016/j.pain.2012.05.017, PMID: 22986070

[ref23] Kwon M. J. Shin H. Y. Cui Y. Kim H. Thi A. H. L. Choi J. Y.. (2015). CCL2 mediates neuron-macrophage interactions to drive proregenerative macrophage activation following preconditioning injury. J. Neurosci. Off. J. Soc. Neurosci. 35, 15934–15947. doi: 10.1523/JNEUROSCI.1924-15.2015, PMID: 26631474 PMC6605453

[ref24] Latremoliere A. Woolf C. J. (2009). Central sensitization: a generator of pain hypersensitivity by central neural plasticity. J. Pain 10, 895–926. doi: 10.1016/j.jpain.2009.06.012, PMID: 19712899 PMC2750819

[ref25] Leypold T. Bonus M. Spiegelhalter F. Schwede F. Schwabe T. Gohlke H.. (2019). N6-modified cAMP derivatives that activate protein kinase a also act as full agonists of murine HCN2 channels. J. Biol. Chem. 294, 17978–17987. doi: 10.1074/jbc.RA119.010246, PMID: 31615893 PMC6879346

[ref26] Li L. Yu T. Yu L. Li H. Liu Y. Wang D. (2016). Exogenous brain-derived neurotrophic factor relieves pain symptoms of diabetic rats by reducing excitability of dorsal root ganglion neurons. Int. J. Neurosci. 126, 749–758. doi: 10.3109/00207454.2015.1057725, PMID: 26441011

[ref27] Liu X.-J. Liu T. Chen G. Wang B. Yu X.-L. Yin C.. (2016). TLR signaling adaptor protein MyD88 in primary sensory neurons contributes to persistent inflammatory and neuropathic pain and neuroinflammation. Sci. Rep. 6:28188. doi: 10.1038/srep28188, PMID: 27312666 PMC4911580

[ref28] Liu D.-L. Lu N. Han W.-J. Chen R.-G. Cong R. Xie R.-G.. (2015). Upregulation of *I*_h_ expressed in IB4-negative Aδ nociceptive DRG neurons contributes to mechanical hypersensitivity associated with cervical radiculopathic pain. Sci. Rep. 5:16713. doi: 10.1038/srep16713, PMID: 26577374 PMC4649360

[ref29] Llorián-Salvador M. Pevida M. González-Rodríguez S. Lastra A. Fernández-García M.-T. Hidalgo A.. (2016). Analgesic effects evoked by a CCR2 antagonist or an anti-CCL2 antibody in inflamed mice. Fundam. Clin. Pharmacol. 30, 235–247. doi: 10.1111/fcp.12182, PMID: 26820818

[ref30] Luo P. Shao J. Jiao Y. Yu W. Rong W. (2018). CC chemokine ligand 2 (CCL2) enhances TTX-sensitive sodium channel activity of primary afferent neurons in the complete Freud adjuvant-induced inflammatory pain model. Acta Biochim. Biophys. Sin. 50, 1219–1226. doi: 10.1093/abbs/gmy123, PMID: 30339176

[ref31] Luo W.-J. Yang F. Yang F. Sun W. Zheng W. Wang X.-L.. (2017). Intervertebral foramen injection of ozone relieves mechanical allodynia and enhances analgesic effect of gabapentin in animal model of neuropathic pain. Pain Physician 20, E673–E685. PMID: 28727712

[ref32] Ma S.-B. Xian H. Wu W.-B. Ma S.-Y. Liu Y.-K. Liang Y.-T.. (2020). CCL2 facilitates spinal synaptic transmission and pain via interaction with presynaptic CCR2 in spinal nociceptor terminals. Mol. Brain 13:161. doi: 10.1186/s13041-020-00701-6, PMID: 33228784 PMC7685578

[ref33] Mizutani K. Roca H. Varsos Z. Pienta K. J. (2009). Possible mechanism of CCL2-induced Akt activation in prostate cancer cells. Anticancer Res. 29, 3109–3113. PMID: 19661323

[ref34] Niemi J. P. DeFrancesco-Lisowitz A. Cregg J. M. Howarth M. Zigmond R. E. (2016). Overexpression of the monocyte chemokine CCL2 in dorsal root ganglion neurons causes a conditioning-like increase in neurite outgrowth and does so via a STAT3 dependent mechanism. Exp. Neurol. 275, 25–37. doi: 10.1016/j.expneurol.2015.09.018 26431741 PMC4688065

[ref35] Qin X. Wan Y. Wang X. (2005). CCL2 and CXCL1 trigger calcitonin gene-related peptide release by exciting primary nociceptive neurons. J. Neurosci. Res. 82, 51–62. doi: 10.1002/jnr.20612, PMID: 16047385

[ref36] Ramesh G. (2014). Novel therapeutic targets in neuroinflammation and neuropathic pain. Inflamm. Cell Signal. 1:e111. doi: 10.14800/ics.111 PMC4457460 26052540

[ref37] Singh S. Anshita D. Ravichandiran V. (2021). MCP-1: function, regulation, and involvement in disease. Int. Immunopharmacol. 101:107598. doi: 10.1016/j.intimp.2021.107598, PMID: 34233864 PMC8135227

[ref38] Sonekatsu M. Taniguchi W. Yamanaka M. Nishio N. Tsutsui S. Yamada H.. (2016). Interferon-gamma potentiates NMDA receptor signaling in spinal dorsal horn neurons via microglia-neuron interaction. Mol. Pain 12:174480691664492. doi: 10.1177/1744806916644927, PMID: 27094552 PMC4956380

[ref39] Song Y. Li H.-M. Xie R.-G. Yue Z.-F. Song X.-J. Hu S.-J.. (2012). Evoked bursting in injured Aβ dorsal root ganglion neurons: a mechanism underlying tactile allodynia. Pain 153, 657–665. doi: 10.1016/j.pain.2011.11.030, PMID: 22237000

[ref40] Tanaka T. Minami M. Nakagawa T. Satoh M. (2004). Enhanced production of monocyte chemoattractant protein-1 in the dorsal root ganglia in a rat model of neuropathic pain: possible involvement in the development of neuropathic pain. Neurosci. Res. 48, 463–469. doi: 10.1016/j.neures.2004.01.004 15041200

[ref41] Tao T. Liu Y. Zhang J. Lai W. Long H. (2022). NGF-induced upregulation of CGRP in orofacial pain induced by tooth movement is dependent on Atp6v0a1 and vesicle release. Int. J. Mol. Sci. 23:1440. doi: 10.3390/ijms231911440, PMID: 36232740 PMC9569904

[ref42] Ulens C. Tytgat J. (2001). Gi-and Gs-coupled receptors up-regulate the cAMP cascade to modulate HCN2, but not HCN1 pacemaker channels. Pflugers Archiv. Eur. J. Physiol. 442, 928–942. doi: 10.1007/s004240100617, PMID: 11680627

[ref43] Wainger B. J. DeGennaro M. Santoro B. Siegelbaum S. A. Tibbs G. R. (2001). Molecular mechanism of cAMP modulation of HCN pacemaker channels. Nature 411, 805–810. doi: 10.1038/35081088 11459060

[ref44] Wang F. Ma S.-B. Tian Z.-C. Cui Y.-T. Cong X.-Y. Wu W.-B.. (2021). Nociceptor-localized cGMP-dependent protein kinase I is a critical generator for central sensitization and neuropathic pain. Pain 162, 135–151. doi: 10.1097/j.pain.0000000000002013, PMID: 32773598

[ref45] Wang X. Wang S. Wang W. Duan J. Zhang M. Lv X.. (2016). A novel intrinsic analgesic mechanism: the enhancement of the conduction failure along polymodal nociceptive C-fibers. Pain 157, 2235–2247. doi: 10.1097/j.pain.0000000000000632, PMID: 27583680 PMC5028159

[ref46] Wang C.-H. Zou L.-J. Zhang Y.-L. Jiao Y.-F. Sun J.-H. (2010). The excitatory effects of the chemokine CCL2 on DRG somata are greater after an injury of the ganglion than after an injury of the spinal or peripheral nerve. Neurosci. Lett. 475, 48–52. doi: 10.1016/j.neulet.2010.03.044 20346391

[ref47] Weng X. Smith T. Sathish J. Djouhri L. (2012). Chronic inflammatory pain is associated with increased excitability and hyperpolarization-activated current (*I*_h_) in C-but not Aδ-nociceptors. Pain 153, 900–914. doi: 10.1016/j.pain.2012.01.019, PMID: 22377439

[ref48] White F. A. Sun J. Waters S. M. Ma C. Ren D. Ripsch M.. (2005). Excitatory monocyte chemoattractant protein-1 signaling is up-regulated in sensory neurons after chronic compression of the dorsal root ganglion. Proc. Natl. Acad. Sci. U. S. A. 102, 14092–14097. doi: 10.1073/pnas.0503496102, PMID: 16174730 PMC1236537

[ref49] Wickenden A. D. Maher M. P. Chaplan S. R. (2009). HCN pacemaker channels and pain: a drug discovery perspective. Curr. Pharm. Des. 15, 2149–2168. doi: 10.2174/138161209788489122, PMID: 19519450

[ref50] Wu X.-B. Jing P.-B. Zhang Z.-J. Cao D.-L. Gao M.-H. Jiang B.-C.. (2018). Chemokine receptor CCR2 contributes to neuropathic pain and the associated depression via increasing NR2B-mediated currents in both D1 and D2 dopamine receptor-containing medium spiny neurons in the nucleus accumbens shell. Neuropsychopharmacology 43, 2320–2330. doi: 10.1038/s41386-018-0115-8, PMID: 29993042 PMC6135748

[ref51] Xie R.-G. Chu W.-G. Hu S.-J. Luo C. (2018a). Characterization of different types of excitability in large somatosensory neurons and its plastic changes in pathological pain states. Int. J. Mol. Sci. 19:161. doi: 10.3390/ijms19010161, PMID: 29303989 PMC5796110

[ref52] Xie R.-G. Gao Y.-J. Park C.-K. Lu N. Luo C. Wang W.-T.. (2018b). Spinal CCL2 promotes central sensitization, Long-term potentiation, and inflammatory pain via CCR2: further insights into molecular, synaptic, and cellular mechanisms. Neurosci. Bull. 34, 13–21. doi: 10.1007/s12264-017-0106-5, PMID: 28265898 PMC5587365

[ref53] Yan Y. Zhu M. Cao X. Xu G. Shen W. Li F.. (2023). Thalamocortical circuit controls neuropathic pain via up-regulation of HCN2 in the ventral posterolateral thalamus. Neurosci. Bull. 39, 774–792. doi: 10.1007/s12264-022-00989-5, PMID: 36538279 PMC10169982

[ref54] Young G. T. Emery E. C. Mooney E. R. Tsantoulas C. McNaughton P. A. (2014). Inflammatory and neuropathic pain are rapidly suppressed by peripheral block of hyperpolarisation-activated cyclic nucleotide-gated ion channels. Pain 155, 1708–1719. doi: 10.1016/j.pain.2014.05.021, PMID: 24861581

[ref55] Yue W. W. S. Yuan L. Braz J. M. Basbaum A. I. Julius D. (2022). TRPV1 drugs alter core body temperature via central projections of primary afferent sensory neurons. elife 11:e80139. doi: 10.7554/eLife.80139, PMID: 35968676 PMC9377796

[ref56] Zhang J. De Koninck Y. (2006). Spatial and temporal relationship between monocyte chemoattractant protein-1 expression and spinal glial activation following peripheral nerve injury. J. Neurochem. 97, 772–783. doi: 10.1111/j.1471-4159.2006.03746.x, PMID: 16524371

[ref57] Zhang H. Ma S.-B. Gao Y.-J. Xing J.-L. Xian H. Li Z.-Z.. (2020). Spinal CCL2 promotes pain sensitization by rapid enhancement of NMDA-induced currents through the ERK-GluN2B pathway in mouse Lamina II neurons. Neurosci. Bull. 36, 1344–1354. doi: 10.1007/s12264-020-00557-9, PMID: 32809188 PMC7674543

[ref58] Zhao R. Pei G.-X. Cong R. Zhang H. Zang C.-W. Tian T. (2014). PKC-NF-κB are involved in CCL2-induced Nav1.8 expression and channel function in dorsal root ganglion neurons. Biosci. Rep. 34:140005. doi: 10.1042/BSR20140005, PMID: 24724624 PMC4062041

