# Supplementary material for: Peripheral CCL2 induces inflammatory pain via regulation of Ih currents in small diameter DRG neurons
Source: Front Mol Neurosci. 2023 Oct 4;16:1144614. doi: 10.3389/fnmol.2023.1144614 (PMC10582564; doi:10.3389/fnmol.2023.1144614)
Supplement: Supplementary file 3 [file Data_Sheet_2.DOCX]

**Supplementary Informations for**

**Peripheral CCL2 induces inflammatory pain via regulation of Ih currents in small diameter DRG neurons**

Lamei Li^1,2, †^, Yuanying Liu^1,2, †^, Wenchao Hu^1, †^, Jing Yang^3^, Suibin Ma^1^, Zhicheng Tian^1^, Zixuan Cao^4^, Kunqing Pan^5^, Ming Jiang^2^, Xia Liu^2^, Shengxi Wu^1*^, Ceng Luo^1*^ and Rou-Gang Xie^1*^

^1^ Department of Neurobiology, School of Basic Medicine, Fourth Military Medical University, Xi’an, China

^2^ School of Life Sciences & Research Center for Resource Peptide Drugs, Shaanxi Engineering & Technological Research Center for Conversation & Utilization of Regional Biological Resources, Yan’an University, Yan’an, China,

^3^ Heart Hospital, Xi’an International Medical Center Hospital, Xi’an, China

^4^ No.6 Cadet Regiment, School of Basic Medical Sciences, Fourth Military Medical University, Xi’an, China

^5^ No.19 Cadet Regiment, School of Basic Medical Sciences, Fourth Military Medical University, Xi’an, China.

^†^ These authors contributed equally.

*** Correspondence:**

Rou-Gang Xie ([rgxie@fmmu.edu.cn](mailto:rgxie@fmmu.edu.cn)); Ceng Luo ([luoceng@fmmu.edu.cn](mailto:luoceng@fmmu.edu.cn)); Shengxi Wu (shengxi@fmmu.edu.cn)

**This PDF file includes:**

Figures. S1 to S4 and legends

**Supplementary figures and legends**


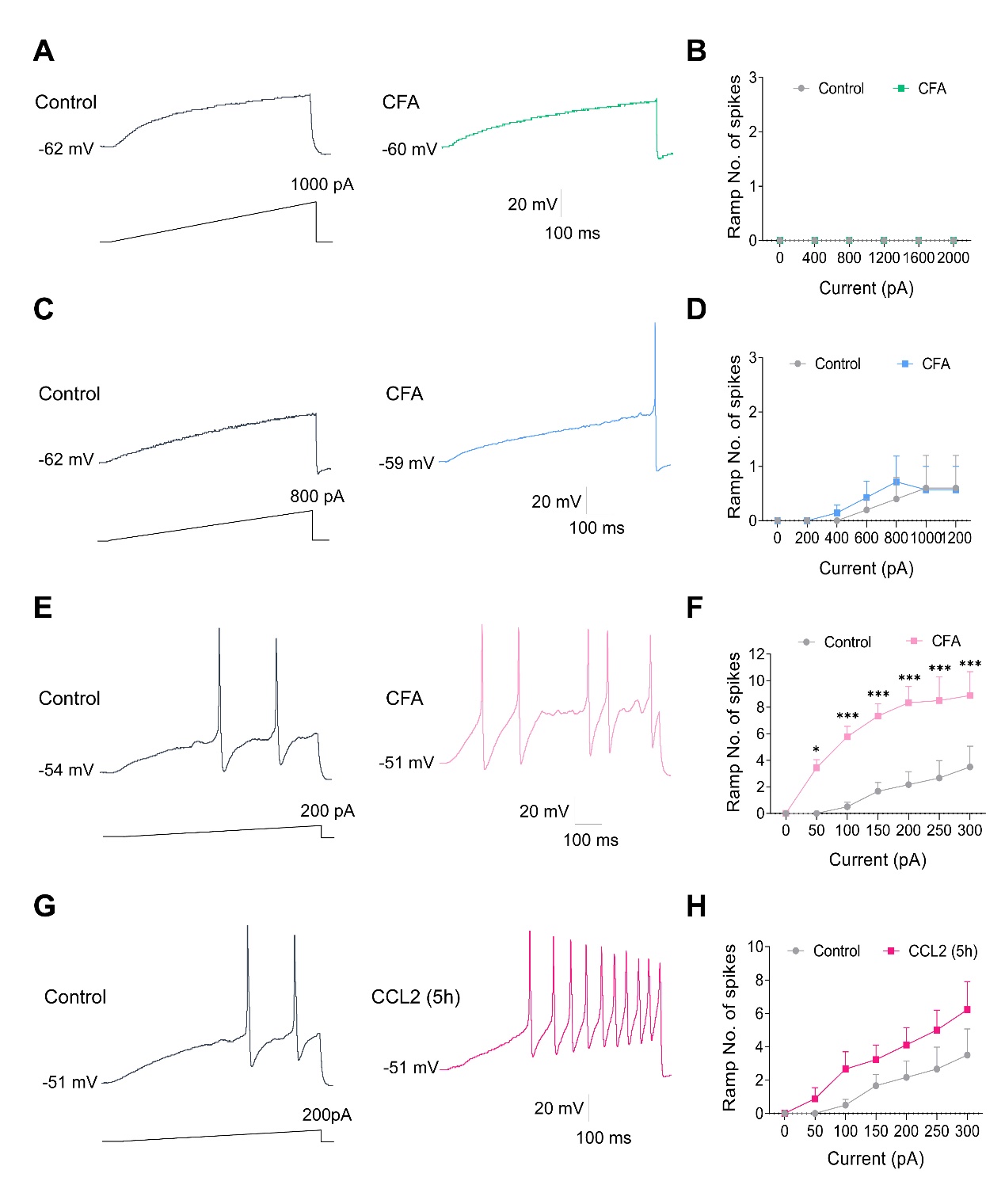


**Fig.S1 CFA and CCL2-induced inflammation increases excitability of small-diameter DRG neurons but not large or medium-diameter DRG neurons.** **(A,B)** Typical traces (A) and showing spike firing in response to ramp current injection at 1000 pA and the I-O curve (B) of large diameter DRG neurons in response to a ramp current injection in both control and CFA-inflamed states (n = 5-7, by two-way ANOVA, Fisher’s LSD test). **(C,D)** Typical traces (C) showing spike firing in response to ramp current injection at 800 pA and the I-O curve (D) of medium diameter DRG neurons in response to a ramp current injection in both control and CFA-inflamed states (n = 6-7, by two-way ANOVA, Fisher’s LSD test). **(E,F)** Typical traces (E) showing spike firing in response to ramp current injection at 200 pA and the I-O curve (F) of small diameter DRG neurons in response to a ramp current injection in both control and CFA-inflamed states (n = 9, by two-way ANOVA, Fisher’s LSD test). **(G,H)** Typical traces (G) showing spike firing in response to ramp current injection at 200 pA and the I-O (H) curve of small diameter DRG neurons in response to a ramp current injection both in control and 5 hours after incubation with CCL2 (n = 8, by two-way ANOVA, Fisher’s LSD test). All data are presented as mean ± SEM. *p < 0.05, **p < 0.01, ***p < 0.001, ****p< 0.0001.


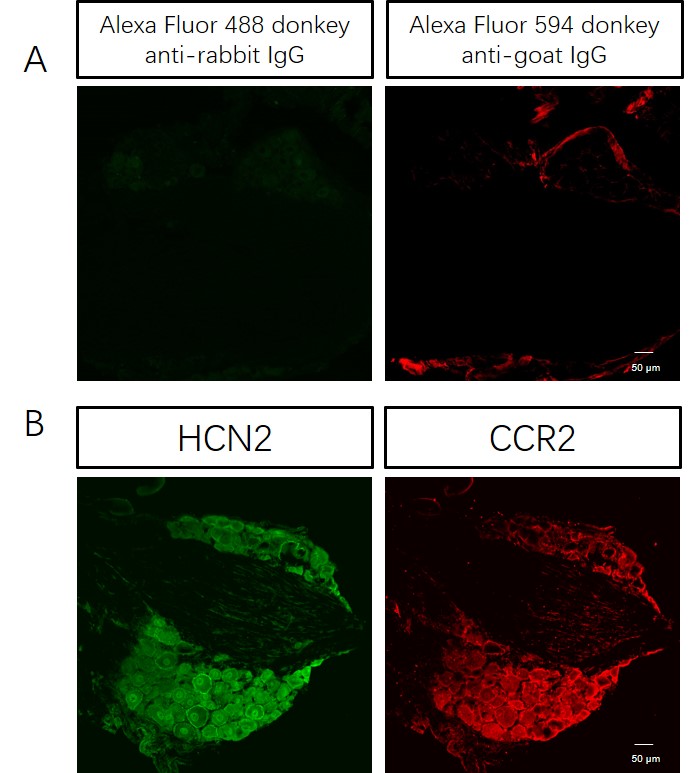


**Fig.S2** **The validity and selectivity of the antibodies. (A)** Immunohistochemical staining map of mouse DRG section was obtained after two nights of antibody dilution staining and direct staining of secondary antibody. **(B)** The DRG sections of mice were taken, and the immunohistochemical staining map of the second antibody was obtained after the first antibody was stained for two nights.


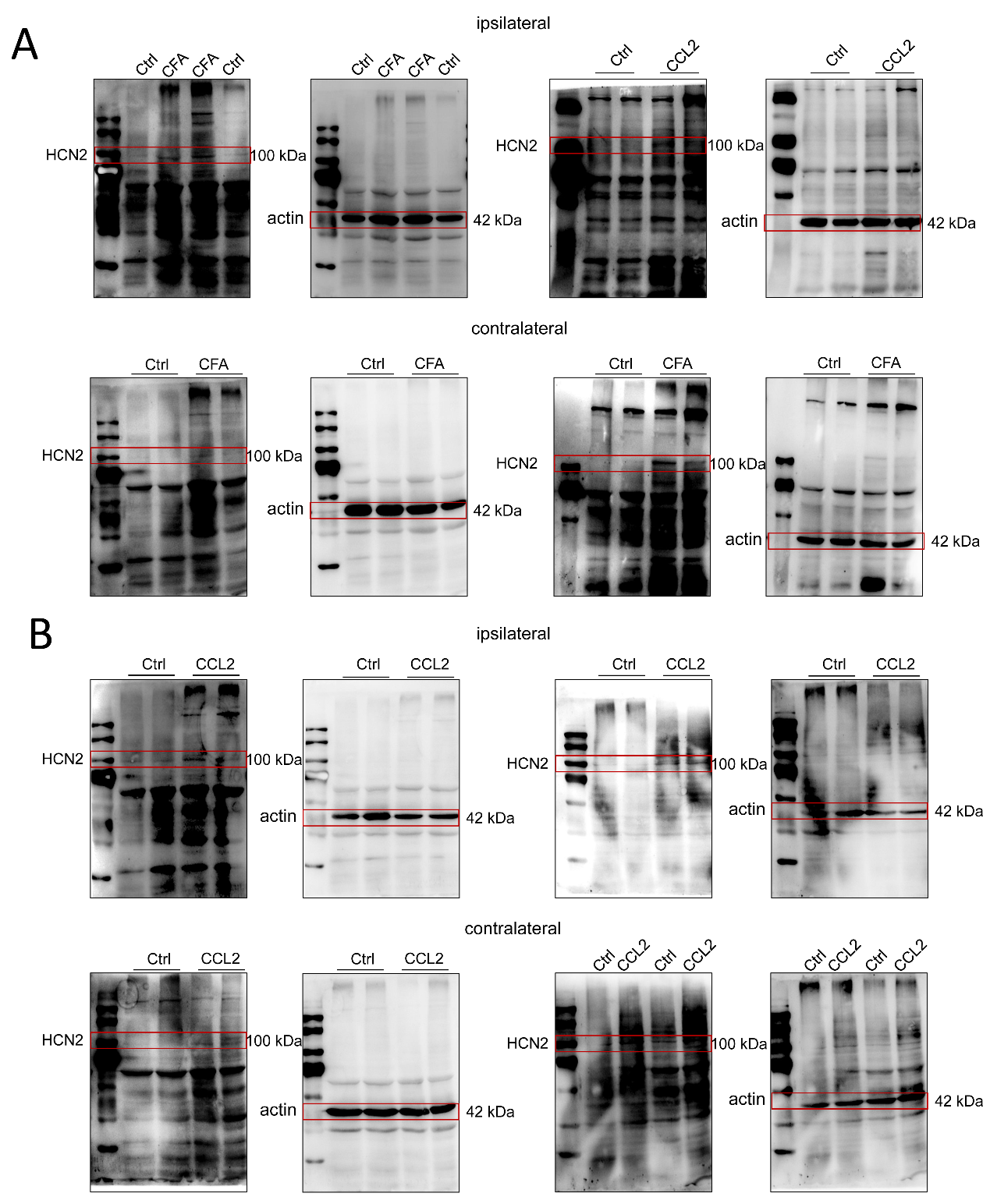


**Fig.S3 Full size gels of Figure 6R,S.** (A) Full size gels of Figure 6R. (B) Full size gels of Figure 6S.


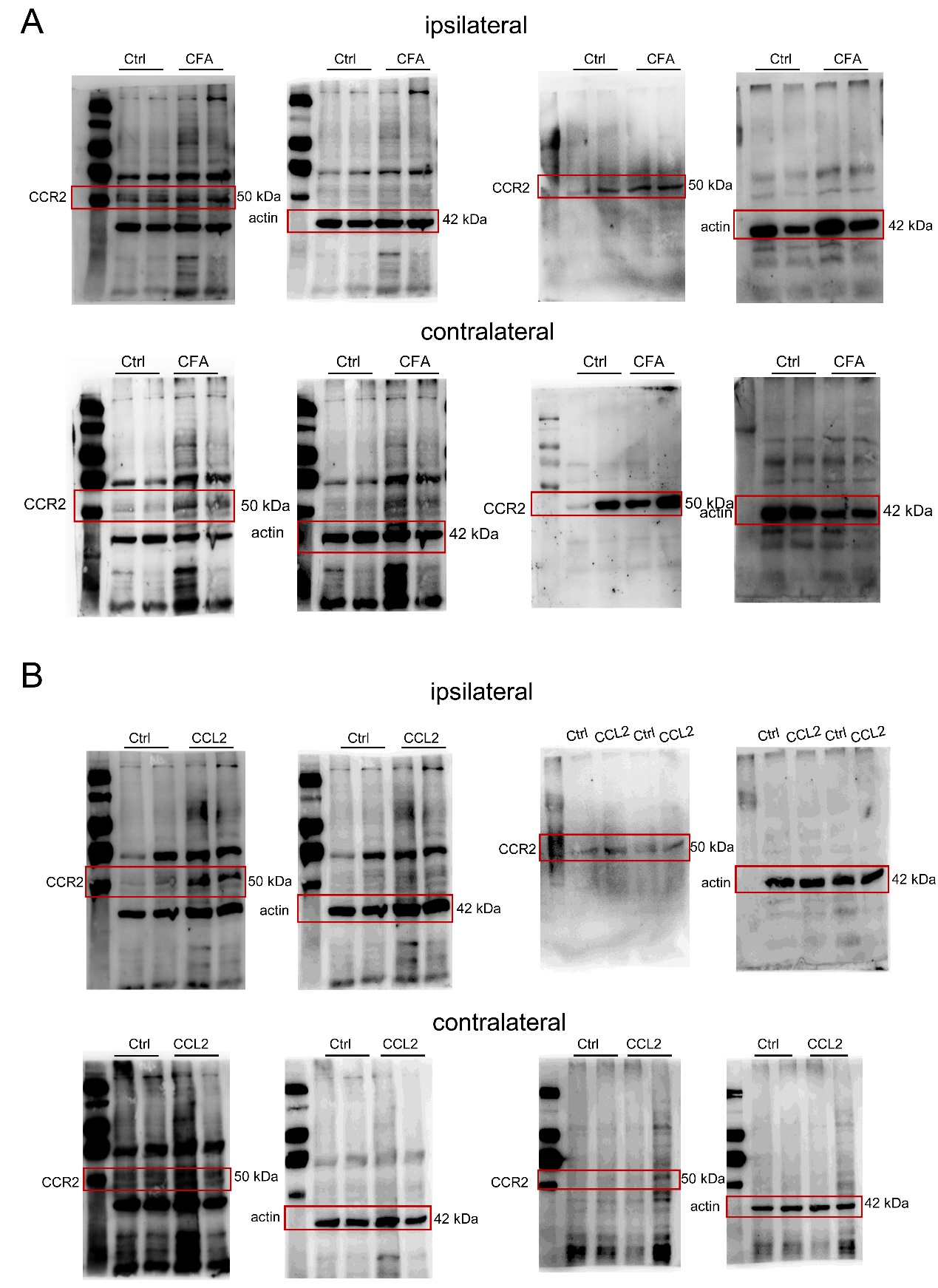


**Fig.S4 Full size gels of Figure 6T,U.** (A) Full size gels of Figure 6T. (B) Full size gels of Figure 6U.
